# Case report: Minimal tissue damage and low coagulation liver resection for hepatoblastoma using indocyanine green fluorescence and water-jet dissector

**DOI:** 10.3389/fped.2023.1221596

**Published:** 2023-07-06

**Authors:** Shun Onishi, Takafumi Kawano, Nanako Nishida, Chihiro Kedoin, Ayaka Nagano, Masakazu Murakami, Koshiro Sugita, Toshio Harumatsu, Mitsuru Muto, Satoshi Ieiri

**Affiliations:** Department of Pediatric Surgery, Research Field in Medical and Health Sciences, Medical and Dental Area, Research and Education Assembly, Kagoshima University, Kagoshima, Japan

**Keywords:** hepatoblastoma, hepatectomy, near-infrared fluorescence imaging, indocyanine green, water-jet dissector, pediatric surgery

## Abstract

Near-infrared (NIR) fluorescence imaging with indocyanine green (ICG) has gained popularity in pediatric surgery as it has in general surgery. In addition, a water-jet dissector (WJD) has been successfully introduced in adult hepatic surgery. Tissue structures are dissected selectively and gently by the WJD. However, there have been no reports of hepatic resection for pediatric patients using a WJD. We applied NIR fluorescence imaging with ICG to visualize the resection line of the liver and used a WJD for liver parenchyma dissection in pediatric hepatoblastoma. The patient was a 3-year-old girl with a large liver tumor. Enhanced computed tomography revealed a liver tumor (maximum diameter: 120 mm) in the right lobe and three small lung metastases. The liver tumor was diagnosed as hepatoblastoma (PRETEXT 2) based on an open biopsy. We performed right hepatectomy after neoadjuvant chemotherapy. The right lobe was mobilized from the diaphragm, and then intraoperative ultrasound was performed to detect the localization of the tumor and its proximity to the vascular structures. We detected the right hepatic artery (RHA), right portal vein (RPV), and right hepatic vein (RHV). The middle hepatic vein was not involved. After ligation of the RHA and RPV to selectively control the right lobe inflow, ICG was administered intravenously and observed by an NIR endoscope. The resection line was clearly visualized by overlaying images in comparison to conventional demarcation line detection. Then, we used a WJD to dissect the parenchyma. Small vessels were divided from parenchymal tissue and were clearly visible. We resected them after clamping with metal clips. Finally, the RHV was transected by a linear stapler, and right hepatectomy was completed with 25 ml of blood loss. There was no postoperative hemorrhage. We performed hepaticojejunostomy because of stricture of the common bile duct on postoperative day 302. The patient was discharged after adjuvant chemotherapy. NIR imaging clearly showed the resection line. The WJD automatically separated, and thus made visible, the more resistant duct and vessel structures from the parenchyma. The combined use of NIR imaging and WJD was useful for pediatric hepatectomy.

## Introduction

The use of near-infrared (NIR) fluorescence imaging with indocyanine green (ICG) has gained popularity in pediatric surgery (1). This technique is often used in hepatectomy for adults and pediatric patients (2, 3). Conventionally, the demarcation line in hepatectomy was grossly distinguished. However, the NIR-ICG technique is now widely used to detect the visible demarcation line.

A water-jet dissector (WJD) has been successfully introduced in hepatic surgery in adult patients (4, 5). The WJD enables tissue structures to be gently and selectively dissected. Blood vessels and nerves remain intact up to a certain pressure. Then, ligation with sutures or vessel sealing using energy devices are used for residual vascular structure depending on their size. While hepatic resection using a WJD has been reported in adult patients, it has not been reported in pediatric patients.

In this study, we applied NIR fluorescence imaging with ICG to visualize the resection line of the liver and used a WJD for dissection of the liver parenchyma in a case of pediatric hepatoblastoma.

## Case report

**Figure 1:**
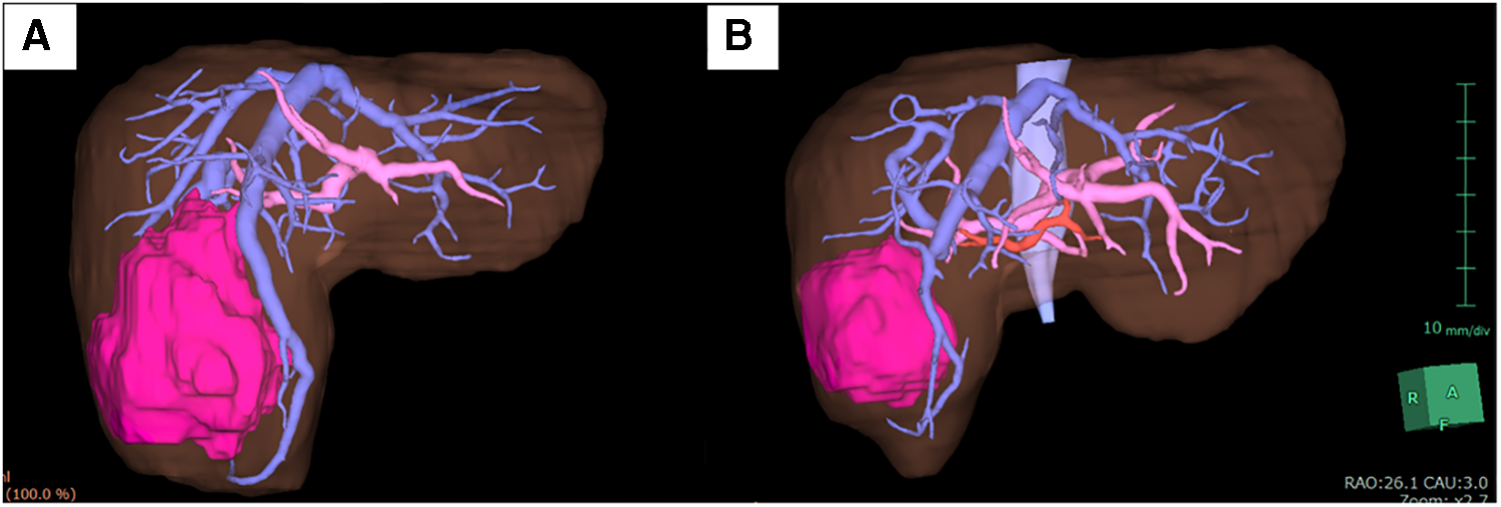
The tumor size. (**A**) Before chemotherapy (120 mm). (**B**) After chemotherapy (45 mm).

A 3-year-old girl with a large liver tumor and elevated alpha-fetoprotein (46,455 ng/ml) was admitted to our institution. Enhanced magnetic resonance imaging and enhanced computed tomography (CT) revealed a liver tumor (maximum diameter: 120 mm) located on the right lobe and three small lung metastases (Figure 1A). The liver tumor was diagnosed as hepatoblastoma (PRETEXT 2) based on an open biopsy. Typical right hepatectomy was planned after neoadjuvant chemotherapy with cisplatin and doxorubicin (according to the International Childhood Liver Tumors Strategy Group, SIOPEL-4 protocol). Neoadjuvant therapy was effective and reduced the size of the liver tumor (from 120 to 45 mm in maximum diameter). In addition, metastatic lesions in the lung showed regression and became invisible on enhanced CT (Figure 1B).

**Figure 2:**
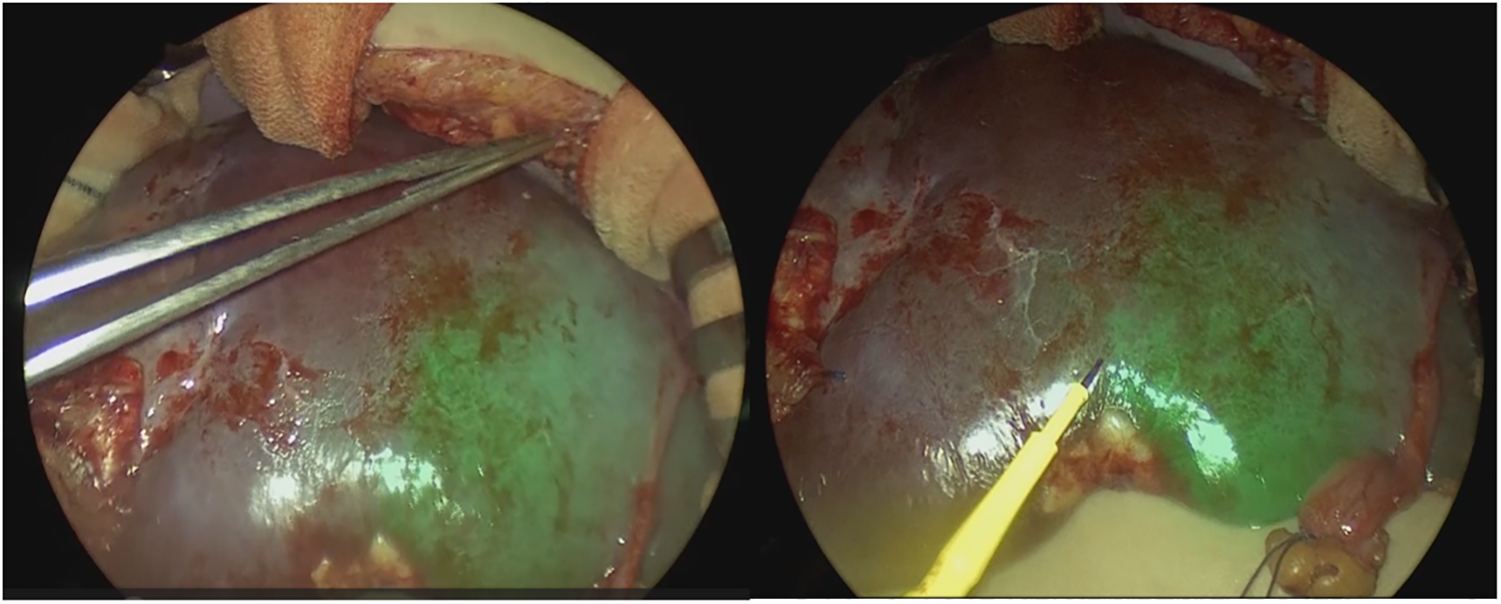
The resection line was visualized by overlaying images.

**Figure 3:**
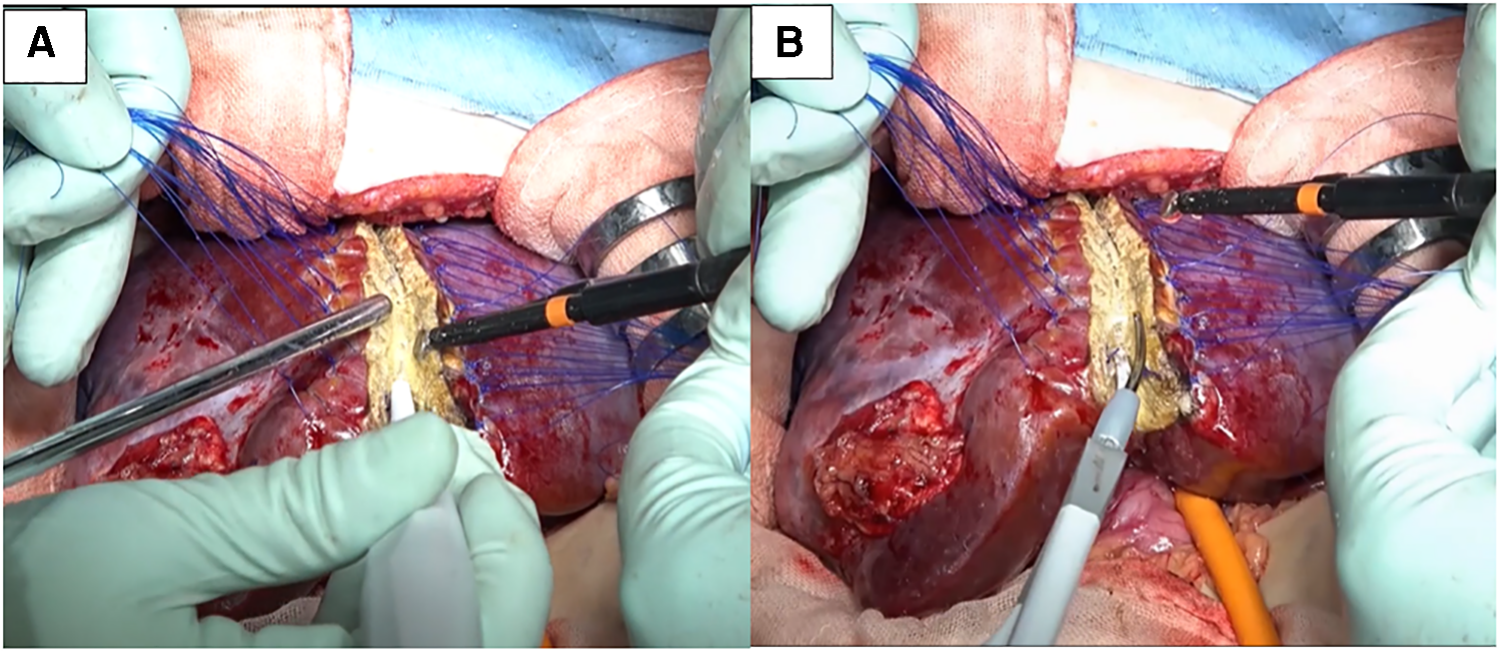
(**A**) We used a water-jet dissector for dissection of the parenchyma. (**B**) Vessels >5 mm were resected with an ultrasonically activated device after division using a water-jet dissector.

Under general and epidural anesthesia, the patient was placed in the supine position. A transverse skin incision was made in the right subcostal region, and the incision was placed along (and extended) a previous open biopsy scar. The liver was carefully mobilized by dividing the ligamentous attachments to the diaphragm and dividing the falciform ligament. Intraoperative ultrasound was used to detect the localization of the tumor and the distance from vascular structures, especially the middle hepatic vein (MHV). The tumor did not involve the MHV. We carefully divided and ligated the right hepatic artery (RHA), right portal vein (RPV), and right hepatic vein (RHV). After ligation of the RHA and RPV to selectively control the right lobe inflow, ICG [0.5 mg/kg (DIAGNOGREEN, Daiichi Sankyo, Tokyo, Japan)] was administered intravenously and observed by NIR endoscopy (IMAGE1 S 4U RUBINA system; KARL STORZ, Tuttlingen, Germany). The resection line was clearly visualized by overlaying images (Figure 2, Supplementary Video). After placing traction sutures using 3–0 Prolene on both sides of the dissection line, the surface of the liver was coagulated and dissected using an ultrasonically activated device (Harmonic Focus, Ethicon, NJ, United States). When dissecting the deep liver parenchyma, we used a WJD (erbe JET2, Erbe Elektromedizin GmbH, Tübingen, Germany). Small vessels were divided from the parenchymal tissue (Figure 3, Supplementary Video). We resected vessels of >5 mm with an ultrasonically activated device (Harmonic Focus, Ethicon, NJ, United States) after suture ligature or metal clip application. The RHV and Glisson's sheath near the porta hepatis were transected using a linear stapler (Powered ECHELON FLEX® 7, Ethicon). We confirmed that there was no bile leakage from the resection surface of the cross-section of the liver with an NIR camera. A fibrinogen combined sheet (TachoSil, CSL Behring, PA, United States) was applied on the resection surface. A drainage tube was inserted under the right diaphragm. The intraoperative blood loss was 25 ml. There was no postoperative hemorrhage. After hepatectomy, continuous biliary leakage from the extrahepatic bile duct was recognized, and repair surgery and stenting intervention were unsuccessful. Therefore, we performed open hepaticojejunostomy due to stricture of the common bile duct on postoperative day 302. The patient was discharged after adjuvant chemotherapy. Lung metastases were curatively treated with chemotherapy. There were no signs of recurrence at 15 months after the operation.

## Discussion

Hepatoblastoma is the most common liver malignancy in childhood. Chemotherapy including cisplatin is effective, and adjuvant chemotherapy is usually given after tumor resection. However, complete tumor resection remains the best way to achieve a cure and leads to excellent oncologic outcomes. Pediatric surgeons must perform perfect hepatectomy, ensuring negative surgical margins without injuring the hepatic vessels or common hepatic duct.

NIR fluorescence imaging with ICG has emerged as a surgical technique that can be used to assist with the localization of pulmonary metastases secondary to hepatoblastoma. Visualization of hepatoblastoma cells by NIR-ICG has been reported during the exploration of metastatic lesions of the tumor. Souzaki et al. (6) reported the detection of primary tumors in four cases and lung metastasis of hepatoblastoma in six cases. The size of the metastatic tumor they could detect was 7.4 ± 4.1 mm (1.2–15 mm). The depth of the ICG-positive tumors from the lung surface was 0.9 ± 1.9 mm (0–6 mm), and the depth of the single ICG-negative tumor was 12 mm. The NIR-ICG technique can detect small and deep metastatic lesions of hepatoblastoma in the lung. Whitlock et al. (3) reported 14 patients who were treated for liver tumors and concluded that the intraoperative usage of NIR-ICG imaging during partial hepatectomy enhanced identification and guided surgical resection of extrahepatic disease. This technique is practical and safe for hepatectomy, even for pediatric patients.

Conventionally, the resection line of the liver has been detected by the change in the surface color of the liver after ligation of the portal vein and hepatic artery. Bleeding from the resection surface is one of the most common complications of liver resection. After ligation of the portal vein and hepatic artery, we could more clearly detect the resection line with NIR fluorescence imaging with ICG in comparison to conventional confirmation based on the surgeon's macroscopic view. The Tokyo 2020 terminology of liver anatomy and resection (7) suggests that the intersegmental planes between segments must be visualized by ICG staining (negative/positive) in order to enable precise anatomical segmentectomy.

The WJD is already available for hepatectomy in adult surgery. Some reports have compared the WJD and conventional tissue fracture techniques during the first era of the device (8, 9). The WJD provided less blood loss and less coagulation of the resection surface. Excessive coagulation of liver tissue sometimes leads to late-onset bile leakage of the resection surface. A cavitation ultrasonic surgical aspirator (CUSA) is also widely used for liver resection. Efanov et al. (4) reported a randomized prospective study of the outcomes of using WJD and CUSA for laparoscopic liver resection. They concluded that the WJD and CUSA showed similar efficacy and safety in transection of the liver parenchyma during laparoscopic resection. Liver resection is not a common operation for most pediatric surgeons. Therefore, a pediatric surgeon should choose a device that the surgeon is familiar when performing liver surgery.

NIR imaging and WJD are helpful in pediatric hepatectomy. The combined use of new technology provides much safer and more precise liver surgery for pediatric patients.

## Data Availability

The raw data supporting the conclusions of this article will be made available by the authors, without undue reservation.
